# GYY4137, a Hydrogen Sulfide Donor Modulates miR194-Dependent Collagen Realignment in Diabetic Kidney

**DOI:** 10.1038/s41598-017-11256-3

**Published:** 2017-09-07

**Authors:** AM Sashi Papu John, Sourav Kundu, Sathnur Pushpakumar, Maura Fordham, Gregory Weber, Manas Mukhopadhyay, Utpal Sen

**Affiliations:** 1 Department of Physiology, University of Louisville, School of Medicine, Louisville, KY 40202 United States; 2 Present Address: Institute of Advanced Studies in Science and Technology, Guwahati, Assam India

## Abstract

The relationship between hydrogen sulfide (H_2_S), microRNAs (miRs), matrix metalloproteinases (MMPs) and poly-ADP-ribose-polymerase-1 (PARP-1) in diabetic kidney remodeling remains mostly obscured. We aimed at investigating whether alteration of miR-194-dependent MMPs and PARP-1 causes renal fibrosis in diabetes kidney, and whether H_2_S ameliorates fibrosis. Wild type, diabetic Akita mice as well as mouse glomerular endothelial cells (MGECs) were used as experimental models, and GYY4137 as H_2_S donor. In diabetic mice, plasma H_2_S levels were decreased while ROS and expression of its modulator (ROMO1) were increased. In addition, alteration of MMPs-9, −13 and −14 expression, PARP-1, HIF1α, and increased collagen biosynthesis as well as collagen cross-linking protein, P4HA1 and PLOD2 were observed along with diminished vascular density in diabetic kidney. These changes were ameliorated by GYY4137. Further, downregulated miRNA-194 was normalized by GYY4137 in diabetic kidney. Similar results were obtained in *in vitro* condition. Interestingly, miR-194 mimic also diminished ROS production, and normalized ROMO1, MMPs-9, −13 and −14, and PARP-1 along with collagen biosynthesis and cross-linking protein in HG condition. We conclude that decrease H_2_S diminishes miR-194, induces collagen deposition and realignment leading to fibrosis and renovascular constriction in diabetes. GYY4137 mitigates renal fibrosis in diabetes through miR-194-dependent pathway.

## Introduction

Diabetic nephropathy (DN) is a chronic kidney disease which, if untreated, leads to end-stage renal disease (ESRD) affecting a large number of population worldwide. DN is characterized by changes in both morphology and functions of kidney including glomerular hypertrophy, nephrotic syndrome, albuminuria, hypertension and renal fibrosis^1^. Virtually in every type of chronic kidney disease, glomerular- and tubulointerstitial fibrosis are the inevitable consequence of renal pathology characterized by excessive accumulation of extracellular matrix (ECM)^2^. Further, increased renal arterial resistive index due to excess ECM accumulation in diabetic kidney leads to raise of blood pressure and worsens the disease.

The roles of H_2_S as a potent anti-oxidant, anti-inflammatory and cytoprotective agent in diabetes and other pathophysiological conditions are well established^3–6^. Using a rat model of obstructive nephropathy Song *et al*. have shown that plasma levels of H_2_S as well as H_2_S producing enzyme cystathionine β-synthase (CBS) was significantly reduced in the ureteral obstructed kidney leading to ECM deposition and fibrosis, whereas H_2_S donor inhibited renal fibrosis^7^. We have previously demonstrated similar role of H_2_S in maintaining kidney homeostasis and deficiency of H_2_S leads to renovascular remodeling in diseases such as hypertension and diabetes^8–10^. Others have also reported decrease in plasma H_2_S as well as deficiency in tissue expression of H_2_S producing enzymes in kidney worsens DN^11^. Nevertheless, the precise matrix regulatory mechanisms of H_2_S, and whether H_2_S potentially modulates posttranscriptional regulation of ECM gene expression in DN has yet to be fully defined.

Of the well-known factors, which increase the risk of disease, dysregulation of genetic components contributes significantly to the onset and progression of DN. Of such factors, microRNAs (miRs) are small highly conserved non-coding RNA molecules that regulate post-transcriptional regulation of gene expression. Among the known ~2,500 miRNA by deep sequencing of human genome^12^, miR-192, miR-194, miR-204, miR-215 and miR-216 are highly expressed in kidney than other human tissues^13^. Studies have reported the involvement of miRNA in the regulation of reactive oxygen species (ROS), such as superoxide or hydroxyl molecules in many pathological conditions^14–16^. Besides the association with oxidative stress, the differential expression pattern of miRNAs has also been linked to inflammation, insulin signaling and angiogenesis in diabetes^16, 17^. However, the specific roles of miR-194 in DN are still incompletely understood. Additionally, a clear link between miR-194 and ROS in mediating renovascular fibrosis, and whether H_2_S may impede fibrosis by modulating miR-194 in diabetic kidney is unknown.

The proteinases that are involved in synthesis and degradation of ECM are known as matrix metalloproteinases (MMPs). These zinc-dependent endopeptidases regulate most ECM proteins including collagen and elastin during organogenesis, growth and normal tissue turnover^18, 19^. MMP-9, a member of MMP family, is a major enzyme responsible for remodeling of glomerular ECM in DN with increased synthesis and deposition of matrix metallo-proteins^20^. Unlike MMP-9, interstitial collagenase MMP-13 and membrane type MMP-14 are poorly studied to demonstrate whether these peptidases have significant involvement in diabetic renal matrix turnover. In addition, although it was reported that hyperglycemic conditions increased ROS leading to PARP activation^21^, and PARP-1 deficiency alleviated diabetic kidney disease^22^, the precise molecular mechanism was unknown.

Experimental evidence indicate that in type 1 diabetes model (OVE26), the blockade of hypoxia inducible factor-1 (HIF-1) reduced glomerular hypertrophy, ECM deposition and urinary albumin excretion^23^. Therefore, inhibition of HIF-1 may provide an important mechanism to ameliorate DN. In addition, prolyl 4-hydroxylase (P4HA1) catalyzes the formation of 4-hydroxyproline that is essential for three-dimensional folding of newly synthesized procollagen chains. Further, hydroxylysine is commonly found in collagen and PLOD-2 gene provides instructions in the process of making lysyl hydroxylase, an enzyme responsible for modification of lysine to hydroxylysine. However, the roles of HIF-1, P4HA1 and PLOD2 in diabetic renal remodeling, and whether H_2_S has potential to regulate collagen cross-linking are not clearly understood.

In the present study, we therefore sought to delineate the mechanism of miR-194-mediated collagen realignment and fibrosis in type-1 diabetic kidney and whether H_2_S generating compound, GYY4137 can modulate the remodeling process to ameliorate disease progression.

## Results

### GYY4137 (GYY) treatment increased plasma H_2_S levels and diminished blood glucose levels in diabetic mice

**Figure 1:**
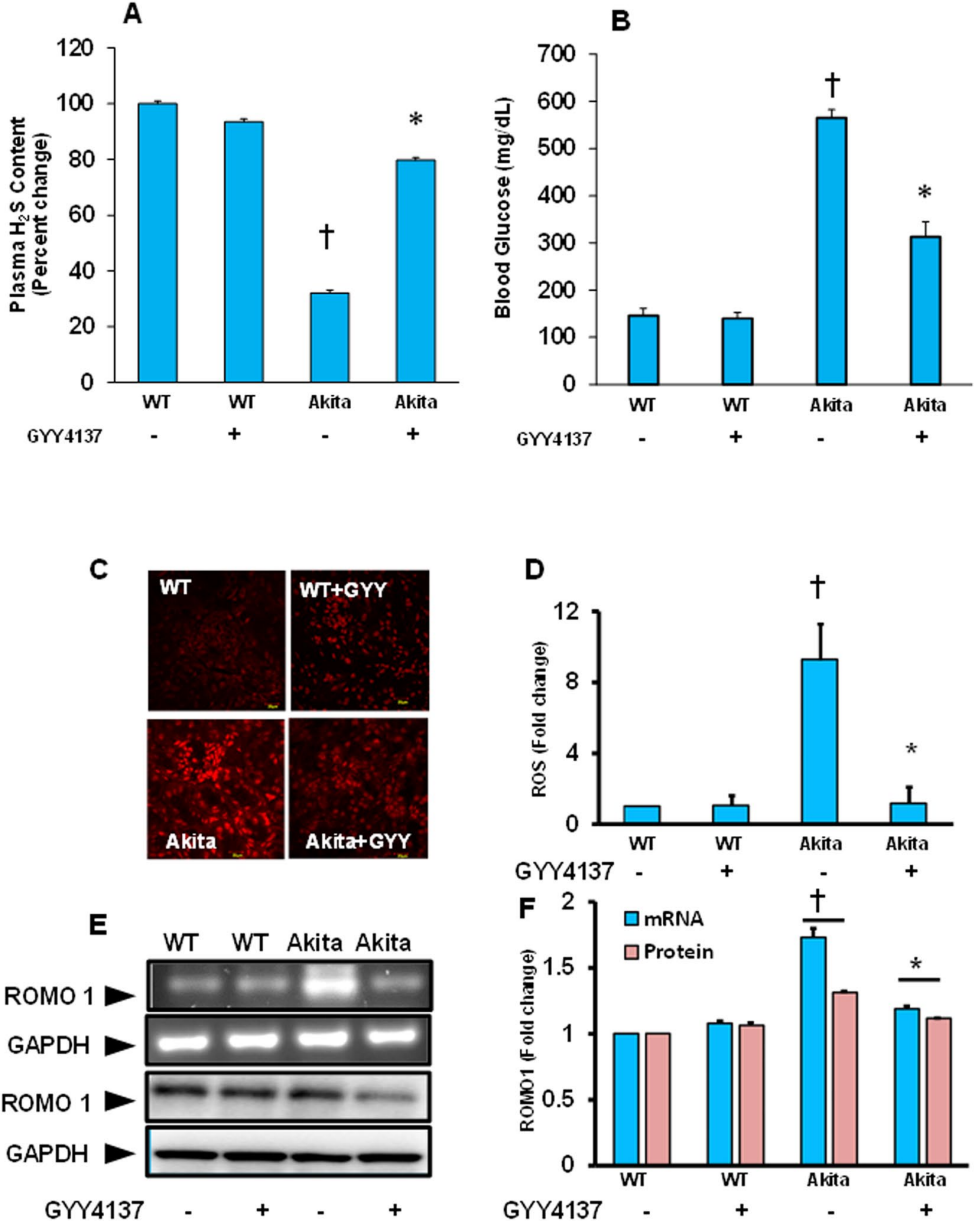
**(A**,**B)** GYY4137 treatment ameliorated increased plasma H_2_S levels (A) and decreased blood glucose **(B)** levels in Akita mice. Plasma H_2_S and blood glucose levels were measured as described in the materials and methods. Data represents mean ± SEM, n = 6–7/ group; ^†^p < 0.05 vs. WT and *p < 0.05 vs. Akita. **(C**–**F)** Increased ROS and ROMO1 in diabetic kidney was mitigated by GYY4137. **(C)** GYY4137 decreased ROS, especially superoxide in diabetic kidney. Renal superoxide was measured by DHE fluorescence activity as described in the material and methods. Scale bar 20 µm. **(D**) GYY4137 also decreased reactive oxygen species modulator 1 (ROMO1), which is a mitochondrial membrane protein responsible for increasing the level of ROS production. ROMO1 mRNA (top) was measured by RT-PCR, and protein expression (bottom) by Western blotting. **(E)** Bar graph represents DHE fluorescence activity which was measured and compared between groups as fold change. **(F)** Bar graphs represent mean fold change of both mRNA and protein expression of ROMO1 using GAPDH as loading control. Data represents mean ± SEM, n = 6–7/ group; ^†^p < 0.05 vs. WT and *p < 0.05 vs. Akita, compared to their respective levels of mRNA and protein expression.

Plasm H_2_S levels were significantly decreased in diabetic Akita mice compared to WT littermates. GYY treatment increased the plasma H_2_S toward normal levels in diabetic mice, which were up to 80% of control level (Fig. 1A). In WT mice with GYY treatment, although a slight decrease of H_2_S levels were detected the difference was non-significant compared to WT without treatment (Fig. 1A). We have earlier reported as well as confirmed in this study that Akita mice have blood glucose levels of ~550 mg/dL around 14–16 weeks of age (Fig. 1B)^9^. To determine the effect of GYY in the glycemic status of Akita mice, we measured blood glucose levels following GYY treatment. The results indicated that GYY treatment lowered blood glucose levels in Akita mice (Fig. 1B). Although the levels were not normalized, the difference in the levels of blood glucose was significant in GYY treated Akita mice compared to non-treated littermates (Fig. 1B). No difference in blood glucose levels were observed in WT control mice without or with GYY treatment.

### GYY4137 (GYY) decreased ROS production and ROMO1 expression in Akita kidney

To measure the levels of reactive oxygen species (ROS), DHE florescence assay was performed in the cryosections as mentioned in the materials and methods. Increased DHE florescence was prominent both in the glomerulus as well as in the tubules of Akita kidney compared to WT kidney (Fig. 1C). Interestingly, Akita mice that have received GYY treatment exhibited normalized DHE florescence, which is similar to WT group (Fig. 1C). On the other hand, although WT with GYY treatment exhibited a slight increase of ROS production compared to WT without GYY, the levels of fluorescence intensity were non-significant. The Bar diagram depicts fold change of ROS production among the groups as quantified by using ImageJ software (Fig. 1D).

In this set of experiment, to determine whether increased ROS production in diabetic kidney was due to altered expression of reactive oxygen species modulator 1 (ROMO1), we performed PCR analysis and Western blotting to measure ROMO1 expression. Results indicated that both the mRNA and protein levels of ROMO1 were significantly increased in Akita kidney compared to WT (Fig. 1E and F). The levels were unaltered in WT groups that were treated without or with GYY. Following GYY treatment in Akita mice, both mRNA and protein levels of ROMO1 were restored toward normal levels similar to WT control (Fig. 1E and F). Together, results presented in the Fig. 1 indicated ROS and mitochondrial membrane protein ROMO1, which is responsible for ROS production in Akita, were increased, and GYY was able to normalize both ROMO1 and ROS in Akita.

### Upregulated expression and activity of MMP-9 was mitigated by GYY4137 (GYY) in diabetic kidney

**Figure 2:**
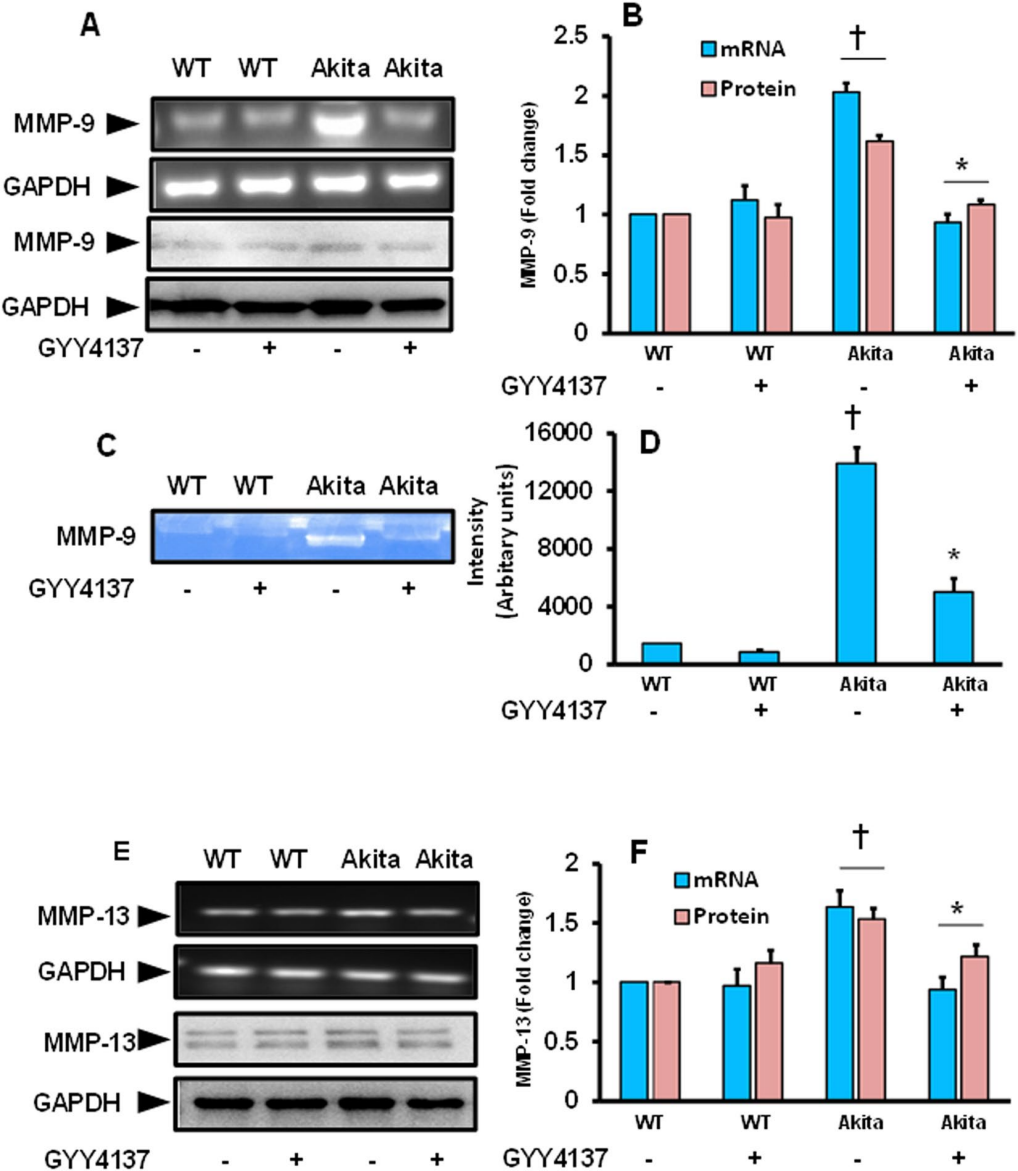
**(A**–**D)** Increased expression and activity of MMP-9 was normalized by GYY4137 in diabetic kidney. **(A)** The mRNA (top) and protein (bottom) expression of MMP-9 was measured by RT-PCR and Western blotting, respectively. **(B)** Bar graph represents mean fold change normalized with GAPDH. **(C)** MMP-9 activity was measured by gelatin zymography as described in the methods. **(D)** Bar graph represents mean fold changes of MMP-9 activity. Data represents mean ± SEM, n = 6–7/ group; ^†^p < 0.05 vs. WT and *p < 0.05 vs. Akita. **(E,F)** Increased expression of MMP-13 was normalized in diabetic kidney following GYY4137 treatment. **(E)** Expression levels of gene (top) and protein (bottom) of MMP-13 in mouse kidney was measured by RT-PCR and Western blotting, respectively. **(F)** Bar graph represents mean fold change normalized with GAPDH. Values are mean ± SEM, n = 6–7/group; ^†^p < 0.05 vs. WT and *p < 0.05 vs. Akita, compared to their respective levels of mRNA and protein expression.

An upregulation of both mRNA and protein levels of MMP-9 were observed in diabetic kidney and these changes were significantly higher compared to WT control (Fig. 2A and B). Akita mice that has received 8 weeks of GYY treatment showed a significant reduction of MMP-9 expression, both at mRNA and protein levels, resembling WT control. No significant differences in MMP-9 mRNA and protein expression were observed in WT mice without or with GYY (Fig. 2A and B).

Results from gelatin zymography showed increased MMP-9 activity in Akita kidney samples (Fig. 2C and D). This increase in both pro- and active MMP-9 in Akita mice was significant in comparison with WT groups, without or with GYY. Protease activity of both pro- and active MMP-9 in Akita mice following GYY treatment was significantly reduced (Fig. 2C and D). There was no significant changes in activity levels between the WT groups, without or with GYY treatment.

### Altered expression of MMP-13 and -14 was normalized by GYY4137 (GYY) treatment

Gene expression and protein levels of MMP-13 were upregulated in Akita mice, and this upregulation was significant compared to WT control (Fig. 2E and F). GYY treatment normalized the expression of both mRNA and protein levels of MMP-13 in Akita mice. No significant changes were observed in WT mice that were treated without or with GYY (Fig. 2E and F).

**Figure 3:**
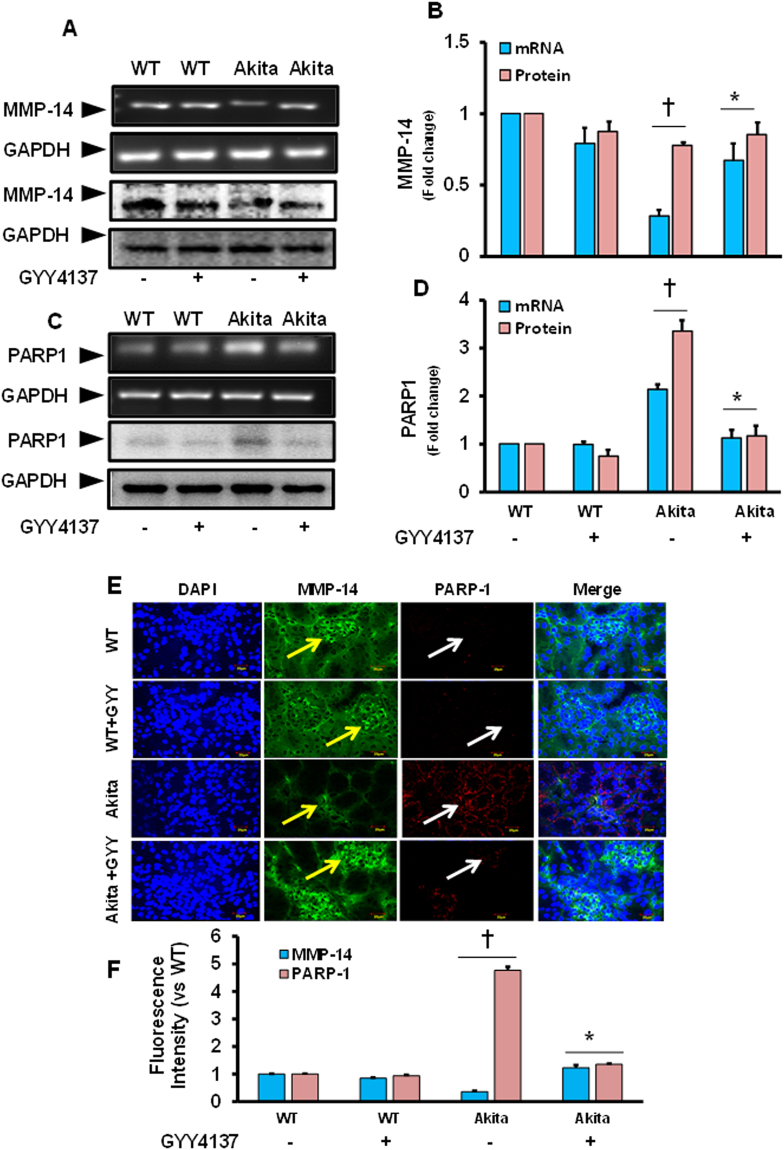
**(A**–**D)** Altered expression of MMP-14 and PARP1 was normalized in diabetic kidney following GYY4137 treatment. **(A)** The mRNA (top) and protein (bottom) expression of MMP-14 in mouse kidney was measured by RT-PCR and Western blotting, respectively. **(B)** Bar graph represents mean fold change of MMP-14 normalized with GAPDH. **(C)** The mRNA (top) and protein (bottom) expression of PARP1 in mouse kidney. **(D)** Bar graph represents mean fold change of PARP1 normalized with GAPDH. Values are mean ± SEM, n = 6–7/group; ^†^p < 0.05 vs. WT and *p < 0.05 vs. Akita. **(E**,**F)** MMP-14 and PARP1 was inversely expressed in the glomerulus and GYY4137 normalized their expression in diabetic kidney. **(E)** Tissue immunostained images of MMP-14 (green; pointed with yellow arrows) and PARP1 (red; pointed with white arrows) in the mouse kidney. Cryosections were incubated overnight with primary antibody and counterstained with appropriate secondary antibodies. Representative images from n = 6–7/group. Original magnification × 60; Scale bar: 20 µm. **(F)** Bar graph represents mean fluorescence intensity changes (fold) vs WT control. Values are mean ± SEM, n = 6–7/group; ^†^p < 0.05 vs. WT and *p < 0.05 vs. Akita, compared to their respective levels of mRNA and protein expression.

Unlike MMPs-9, and -13, mRNA and protein levels of MMP-14 were downregulated in Akita mice, and these were significant compared to WT control (Fig. 3A and B). Interestingly, downregulated MMP-14 mRNA and protein expression was reversed toward normal level in Akita mice following GYY treatment. Of note, expression of MMP-14 both at mRNA and protein level in WT mice remained almost unchanged without or with GYY (Fig. 3A and B).

### Upregulation of PARP-1 in Akita kidney was normalized following GYY4137 (GYY) treatment

Corresponding to the levels of both MMPs-9 and −13, mRNA and protein levels of PARP-1 were significantly upregulated in Akita mice compared to WT controls (Fig. 3C and D). Normalization of PARP-1 levels was observed in diabetic mice after GYY treatment, and this was significant in comparison with Akita mice without GYY. In WT mice that were treated without or with GYY, the expression levels of PARP-1, in both mRNA and protein, remained unchanged (Fig. 3C and D).

### Localization of MMP-14 and PARP-1 in the glomerulus

To determine the localization of MMP-14 and PARP-1 in the kidney tissue, immunostaining was performed following the protocol as described in materials and methods. Results indicated that expression and localization of MMP-14 was high in the glomerulus both in the WT groups treated without or with GYY (Fig. 3E). In Akita mice, although MMP-14 was present in the glomerulus its expression was diminished compared to WT control. Interestingly, GYY treatment increased MMP-14 expression in the glomerulus of Akita mice, which was comparable to WT (Fig. 3E).

Contrary to the MMP-14, the expression of PARP-1 was very low in the glomerulus of WT mice treated without or with GYY, as detected by immunostaining (Fig. 3E). On the other hand, increased expression of PARP-1 was evident in the glomerulus of Akita mice, which was restored to the basal level following GYY treatment (Fig. 3E). The fluorescence intensities of MMP-14 and PARP-1 was quantified using ImageJ software and displayed as bar diagram (Fig. 3F).

### Upregulated collagen and collagen forming molecules were mitigated by GYY4137 treatment in diabetic kidney

**Figure 4:**
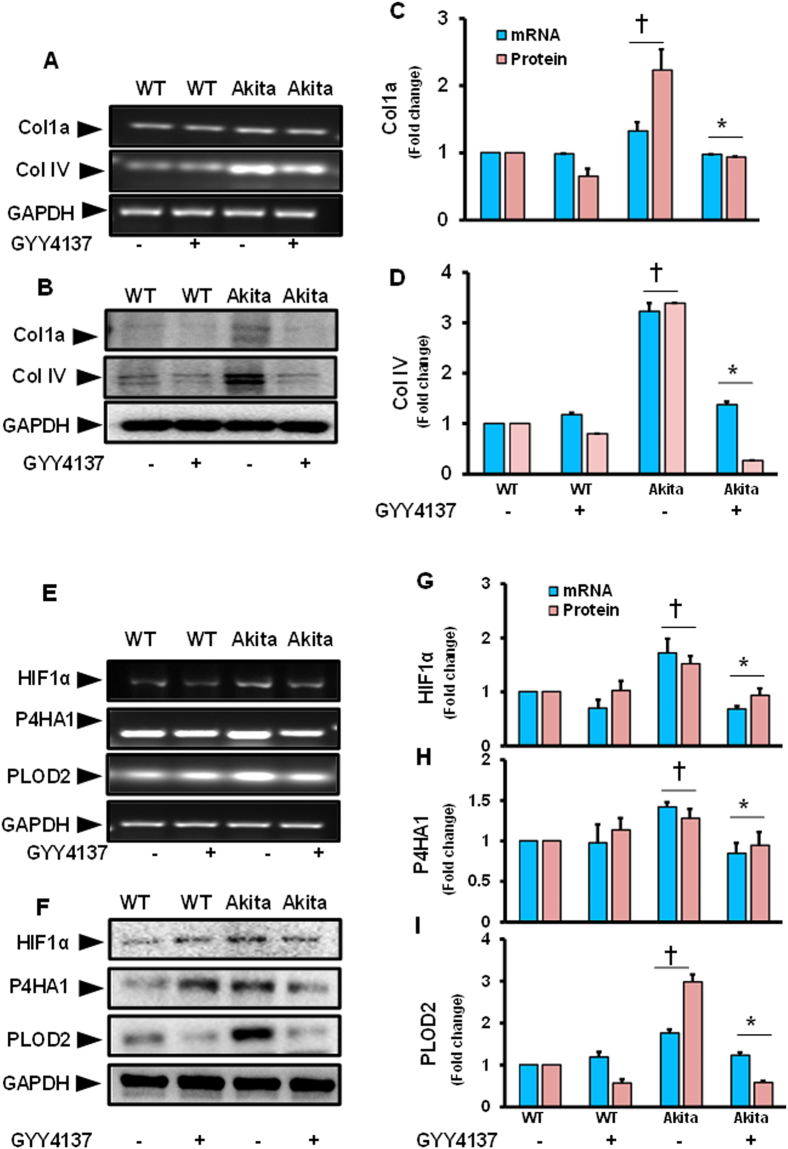
**(A**–**D)** Increased collagen (Col 1a and Col IV) gene and protein expression was mitigated by GYY4137 in diabetic kidney. **(A)** Gene expression of Col 1a and Col IV was measured by RT-PCR and **(B)** protein expression by Western blot analysis. **(C)** and **(D)** Bar graph represents mean fold change of Col1a and Col IV normalized with GAPDH. Values are mean ± SEM, n = 6–7/group; ^†^p < 0.05 vs. WT and *p < 0.05 vs. Akita, compared to their respective levels of mRNA and protein expression. **(E**–**I)** Increased expression of HIF1α, P4HA1 and PLOD2 in diabetic kidney was normalized by GYY4137. **(E)** In the mouse kidney, gene expression of HIF1α, P4HA1 and PLOD2 was measured by RT-PCR, and **(F)** protein expression by Western blotting. **(G**,**H)** and **(I)** are the bar graphs representing mean fold change of HIF1α, P4HA1, and PLOD2 respectively normalized with GAPDH. Values are mean ± SEM, n = 6–7/group; †p < 0.05 vs. WT and *p < 0.05 vs. Akita, compared to their respective levels of mRNA and protein expression.

This experiment was conducted to determine whether the expressions of collagen 1a and IV were changed in Akita mice, and whether GYY has any modulatory roles to prevent the changes. Results indicated that a marked increase in the expression of Col1a and IV, both at mRNA and protein levels, was observed in Akita mice. These changes were significant in comparison with WT control (Fig. 4A,B,C and D). In the Akita mice that received GYY treatment, the expression of both Col1a and IV was suppressed and remained at the basal levels. The reduced levels of both the types of collagen in Akita mice following GYY treatment were significant in comparison with Akita mice without GYY (Fig. 4A,B,C and D). The expression pattern of both Col1a and IV however remained unchanged in WT mice treated without or with GYY (Fig. 4A,B,C and D).

In the following experiment, we focused to study the expression levels of genes as well as their protein that play a pivotal role in collagen formation, i.e., HIF1α, P4HA1 and PLOD2. Following a similar expression trend of Col1a and IV, the expression levels of HIF1α, P4HA1 and PLOD2 in kidney lysates of Akita mice were upregulated at both mRNA and protein levels, and these were significant compared to WT control (Fig. 4E, F,G,H and I). The increased expressions of these collagen forming genes and their protein expression in Akita mice was mitigated and normalized towards the basal levels by GYY treatment (Fig. 4E,F,G,H and I). No significant changes were observed in WT without or with GYY.

### Renal fibrosis and vascular density in Akita kidney was ameliorated with GYY4137 treatment

**Figure 5:**
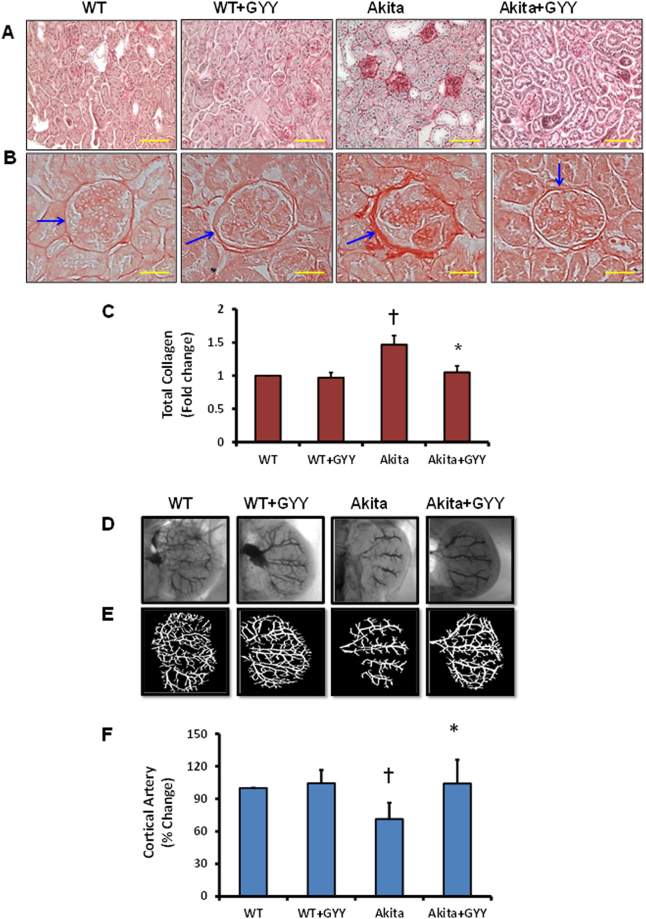
(**A**–**C)** Periglomerular collagen deposition was reduced in diabetic kidney treated with GYY4137. **(A)** Representative photomicrographs of collagen staining with picrosirius red (magnification, × 20). **(B)** Photomicrographs focusing glomerulus to visualize surrounding collagen (blue arrows; magnification × 60. **(C)** Bar graph representing total collagen area against the background, calculated as mean relative intensity in arbitrary unit, and presented as fold change compared to WT. Values are mean ± SEM, n = 6–7/group; †p < 0.05 vs. WT and *p < 0.05 vs. Akita. **(D**–**F)** GYY4137 treatment improved renal vascular perfusion in diabetic kidney. **(D)** Representative Barium x-ray kidney angiograms of all four experimental groups are shown here. **(E)** Vessel segment analysis of angiogram images for all experimental groups. Barium sulfate (50 mM) was infused via infra-renal aorta at constant pressure and time to visualize renal vascular architecture. VesSeg tool was used as described in methods for the analysis of vessel coverage. Total vessel area was calculated using ImageJ software. **(F)** Bar graph represents mean percent change in renal vessel coverage against the background. Values are mean ± SEM, n = 6–7/group; ^†^p < 0.05 vs. WT and *p < 0.05 vs. Akita.

Since we observed increased expression of collagen mRNA and protein in the Akita kidney in our experiment as shown in Fig. 4A,B,C and D, we measured collagen deposition in the kidney, and determined whether GYY has any collagen modulatory roles in the kidney. Results indicated that collagen deposition surrounding the glomerulus was increased in Akita kidney as determined by picrosirius red staining (Fig. 5A and B). Image analysis indicated the accumulation of collagen surrounding the glomerulus was increased significantly in Akita mice compared to WT controls (Fig. 5C). GYY treatment to Akita mice prevented the amount of collagen deposition in the glomerulus, which was similar to the basal level as observed in WT control groups (Fig. 5A, B and C). In the glomerulus of WT mice treated with GYY, the collagen accumulation remained unchanged as compared to WT mice without GYY (Fig. 5A, B and C).

To determine vascular remodeling due to changes in matrix protein and their endogenous modulator such as MMPs, we measured renal vascular densities by Barium sulfate angiography. The obtained images were analyzed by special software as mentioned in the methods. Results indicated that basal level of vascular density in WT mice remained unaltered whether or not treated with GYY (Fig. 5D and E). On the contrary, decreased vascular density was evident in Akita mice. GYY treatment improved the renal vascular density in Akita mice following GYY treatment, which was similar to WT mice (Fig. 5D and E). Bar graphs depict the percent change of renal cortical artery density among the groups (Fig. 5F). Further analysis indicated decreased vascular percentage in Akita mice, which was at the level of 65% in comparison with WT (100%). Interestingly, GYY treatment in Akita mice showed a marked improvement in the cortical arteries densities at the percentage level suggesting better perfusion of renal vasculature following GYY treatment (Fig. 5F).

### Downregulated miR-194 expression in diabetes was normalized by GYY4137 (GYY) treatment

**Figure 6:**
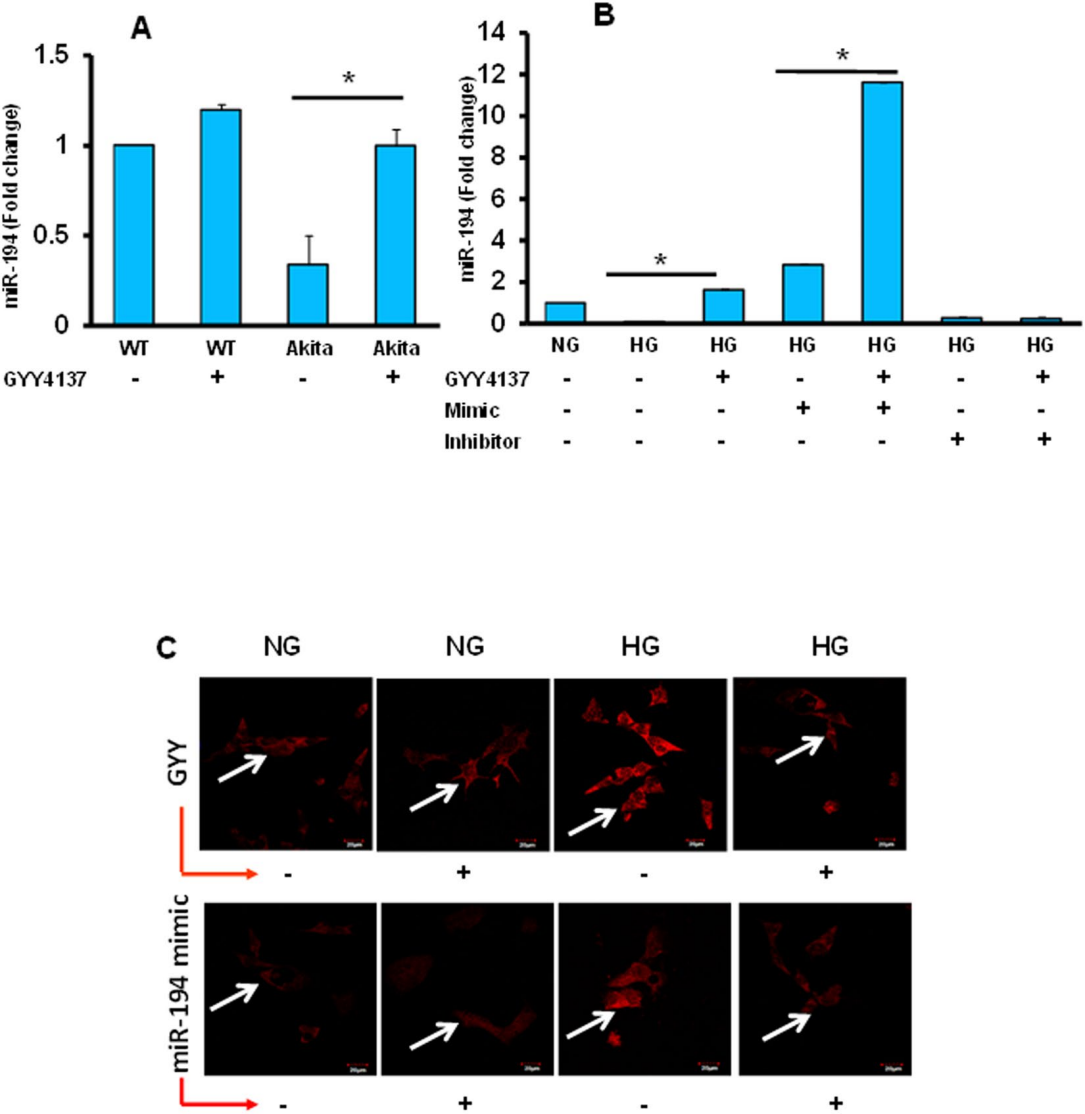
**(A**,**B)** Increased miR-194 expression in diabetic condition was normalized by GYY4137 treatment. The bar diagram represents expression of miR-194 in mouse kidney **(A)** and in mouse glomerular endothelial cells (MGECs) **(B)**. The expression of miR-194 was decreased in both diabetic kidney tissue as well as in hyperglycemic (HG) conditions. The decreased levels of miR-194 in diabetic kidney was normalized with GYY4137 treatment **(A)**, and in MGECs transfected with miR-194 mimic **(B)**. RT-qPCR was performed to measure miR-194. WT and Akita mice were treated without or with GYY4137 as described in the methods. MGECs were transfected with miR-194 mimic and miR-194 inhibitor for 72 hours. Bar graph represents mean relative expression as fold change ± SEM, *p < 0.05 between groups; n = 6–7/ group or independent experiments. **(C)** Increased ROS in HG condition was diminished by either GYY4137 or miR-194 mimic treatment in MGECs. GYY4137 decreased ROS, especially superoxide in HG condition in MGECs (top panel). Similar effect was observed with miR-194 mimic treatment (bottom panel). Superoxide was measured by DHE fluorescent activity as described in the material and methods. Representative images are from n = 5 independent experiments.

In the present study, we first measured miR-194 expression in kidney tissues as well as in MGECs, and the results were summarized in Fig. 6A and B. The qPCR results from kidney lysate of Akita mice clearly indicated low levels of miR-194 compared to WT control (Fig. 6A). The decreased levels of miR-194 in Akita kidney was significant compared to both WT control and WT mice treated with GYY (Fig. 6A). The levels of miR-194 were restored to normal range in Akita mice treated with GYY, which was significant when compared to Akita mice without GYY. No significant changes were observed between the WT groups treated without or with GYY (Fig. 6A).

Results obtained from MGECs experiment following HG condition and transfected with both mimic and inhibitor of miR-194 for 72 hours were compiled in Fig. 6B. In HG cells, miR-194 was significantly decreased compared to normoglycemic (NG) condition, and it was increased following GYY treatment (Fig. 6B). Similar result was obtained in HG cells in the presence of miR-194 mimic. Interestingly, HG cells treated with GYY in the presence of mimic demonstrated even higher and robust increase of miR-194 levels compared to HG, HG plus GYY and HG plus mimic (Fig. 6B). On the other hand, HG cells that was treated without or with GYY in the presence of inhibitor showed a significant reduction in miR-194 expression compared to NG, and these results were comparable to HG alone. Overall, the expression of miR-194 in HG cells was 7-fold higher in the presence of mimic and GYY treatment than the HG cells treated only with GYY (Fig. 6B).

### In HG condition miR-194 mimic or GYY4137 (GYY) mitigated ROS production in MGECs

We observed downregulation of miR-194 and GYY treatment normalized miR-194 expression in both *in vivo* and *in vitro* hyperglycemic (HG) condition (Fig. 6A and B). We also observed GYY diminished ROS production in diabetic Akita kidney (Fig. 1C and D). To determine a relationship between miR-194 and H_2_S in ROS production, we treated MGECs without or with GYY or miR-194 mimic in HG condition, and measured ROS by DHE. Results indicated that miR-194 mimic mitigated superoxide (ROS) production in HG condition (Fig. 6C). Cells in NG condition without or with miR-194 mimic had basal level of DHE fluorescence indicating NG cells are not in stress (Fig. 6C). Similarly, when we treated HG cells with GYY, the superoxide production was diminished compared to non-treated cells (Fig. 6C); whereas fluorescence intensity remained at the basal level under NG condition irrespective of whether or not the cells were treated with GYY. These results indicate that ROS production in HG condition was in part, mediated by H_2_S and miR-194, and H_2_S regulates miR-194 in HG condition.

### Transfection of miR-194 mimic normalized altered expression of ROMO-1, MMP-9, -13 and -14 in hyperglycemic (HG) MGECs

**Figure 7:**
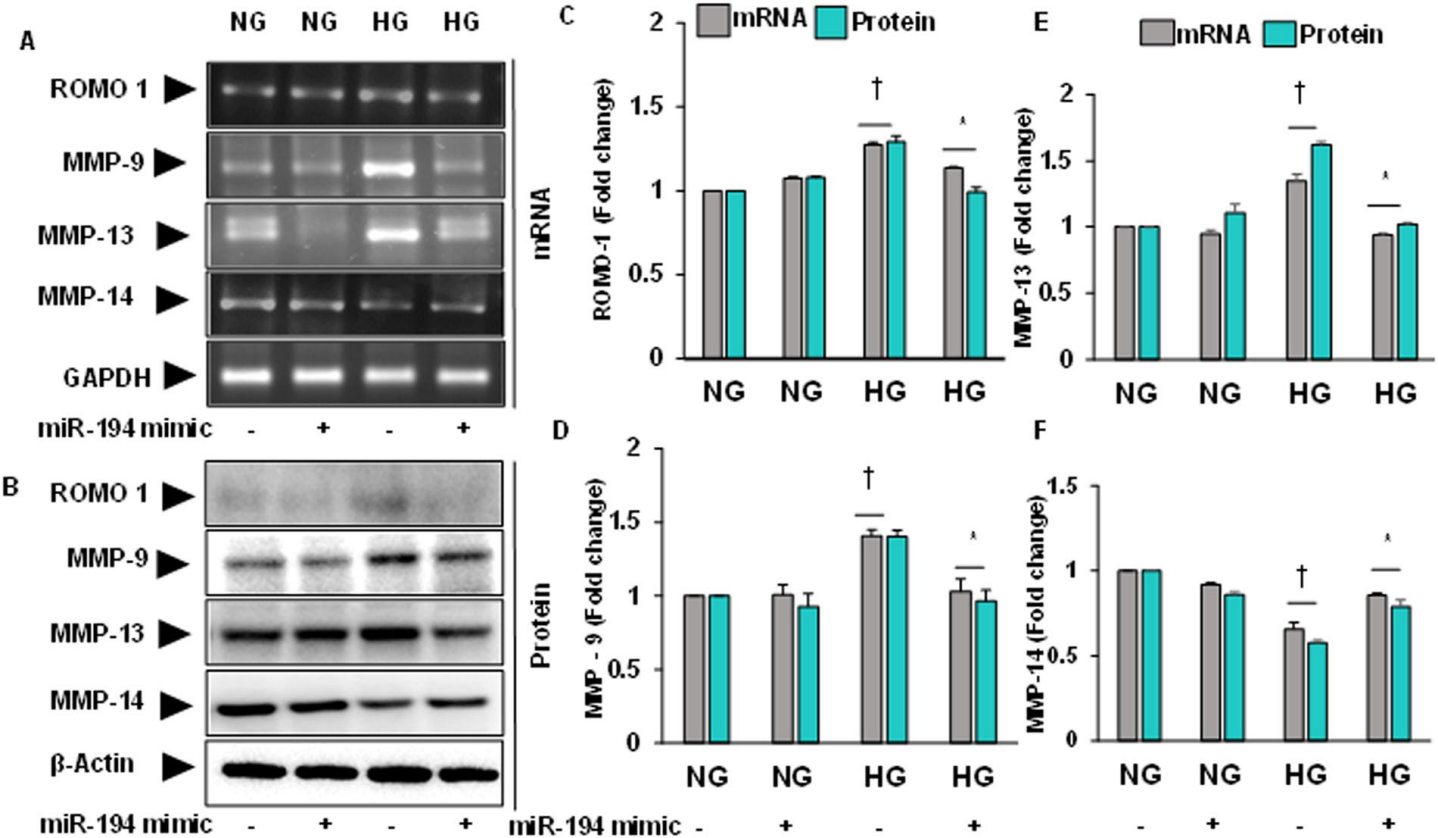
Altered ROMO1, MMP-9, -13 and -14 mRNA and protein expression was ameliorated by miR-194 mimic in HG condition. **(A)** mRNA expression was measured by RT-PCR, and **(B)** protein expression by Western blotting. **(C**,**D**,**E)** and **(F)** Bar graphs represent mean fold changes of ROMO1, MMP-9, -13 and -14 respectively normalized with GAPDH / β-actin. Values are mean ± SEM, n = 5 independent experiments; ^†^p < 0.05 vs. NG alone and *p < 0.05 vs. HG alone; compared to their respective levels of mRNA and protein expression.

Since miR-194 was downregulated in diabetic condition (Fig. 6A and B) and miR-194 mimic decreased ROS production in HG condition (Fig. 6C), we determined whether miR-194 regulates ROMO1 and MMPs under *in vitro* HG condition using MGECs. Our results indicated that ROMO1, MMP-9, -13 were upregulated and MMP-14 was downregulated in HG compared to NG condition, both at transcriptional and protein levels (Fig. 7A,B,C,D,E and F). Interestingly, miR-194 mimic transfection reversed these changes in HG condition compared to NG control transfection. No significant changes, in terms of mRNA and protein expression of these molecules, were observed in NG condition without or with miR-194 mimic transfection (Fig. 7A,B,C,D,E and F). These results suggest that miR-194 regulates ROMO1, MMP-9, -13 and -14 in HG condition *in vitro*.

### miR-194 mimic normalized PARP-1, Col1a and Col IV expression in HG condition

**Figure 8:**
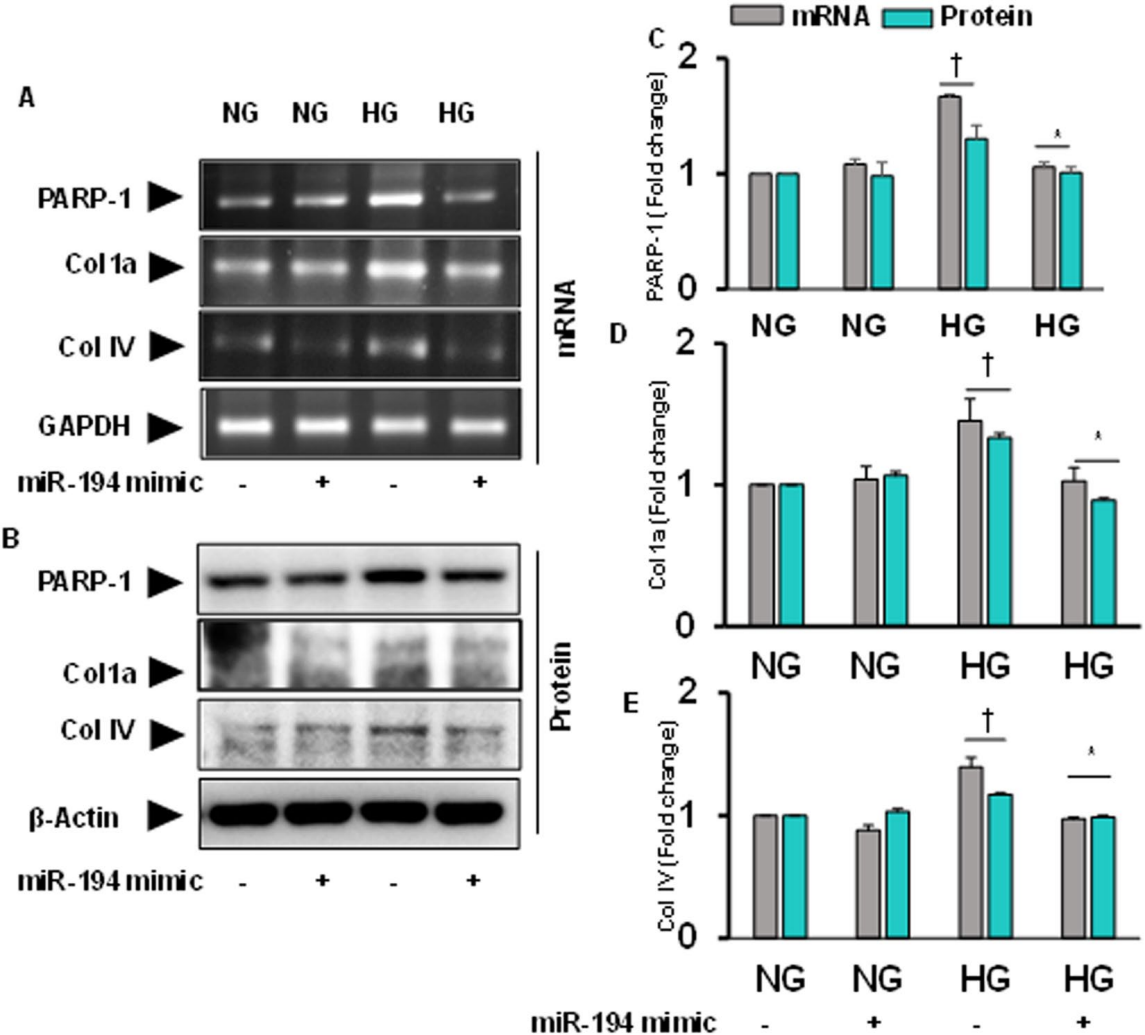
Increased PARP-1, Col 1a and Col IV transcript and protein expression was mitigated by miR-194 mimic transfection in HG condition. **(A)** Gene expression of PARP-1, Col 1a and Col IV was measured by RT-PCR and **(B)** protein expression by Western blotting. **(C,D)** and **(E)** Bar graphs represent mean fold change of PARP-1, Col1a and Col IV respectively normalized with GAPDH / β-actin. Values are mean ± SEM, n = 5 independent experiments; †p < 0.05 vs. NG alone and *p < 0.05 vs. HG alone; compared to their respective levels of mRNA and protein expression.

In these sets of experiment, we determined whether miR-194 mimic could modulate expression of PARP-1, Col1a and Col IV in HG condition. Under *in vitro* conditions, we observed increased expression of these molecules in HG condition compared to NG condition in MGECs (Fig. 8A,B,C,D and E). When MGECs were transfected with miR-194 mimic, the expression of these molecules were normalized in HG condition. No significant changes were observed in cells without or with miR-194 mimic transfection in NG condition (Fig. 8A,B,C,D and E). These results suggest that miR-194 regulates PARP-1, Col1a and Col IV in MGECs under HG condition.

### Altered expression of HIF1α, PLOD2 and P4HA1 in HG condition was ameliorated by miR-194 mimic transfection

**Figure 9:**
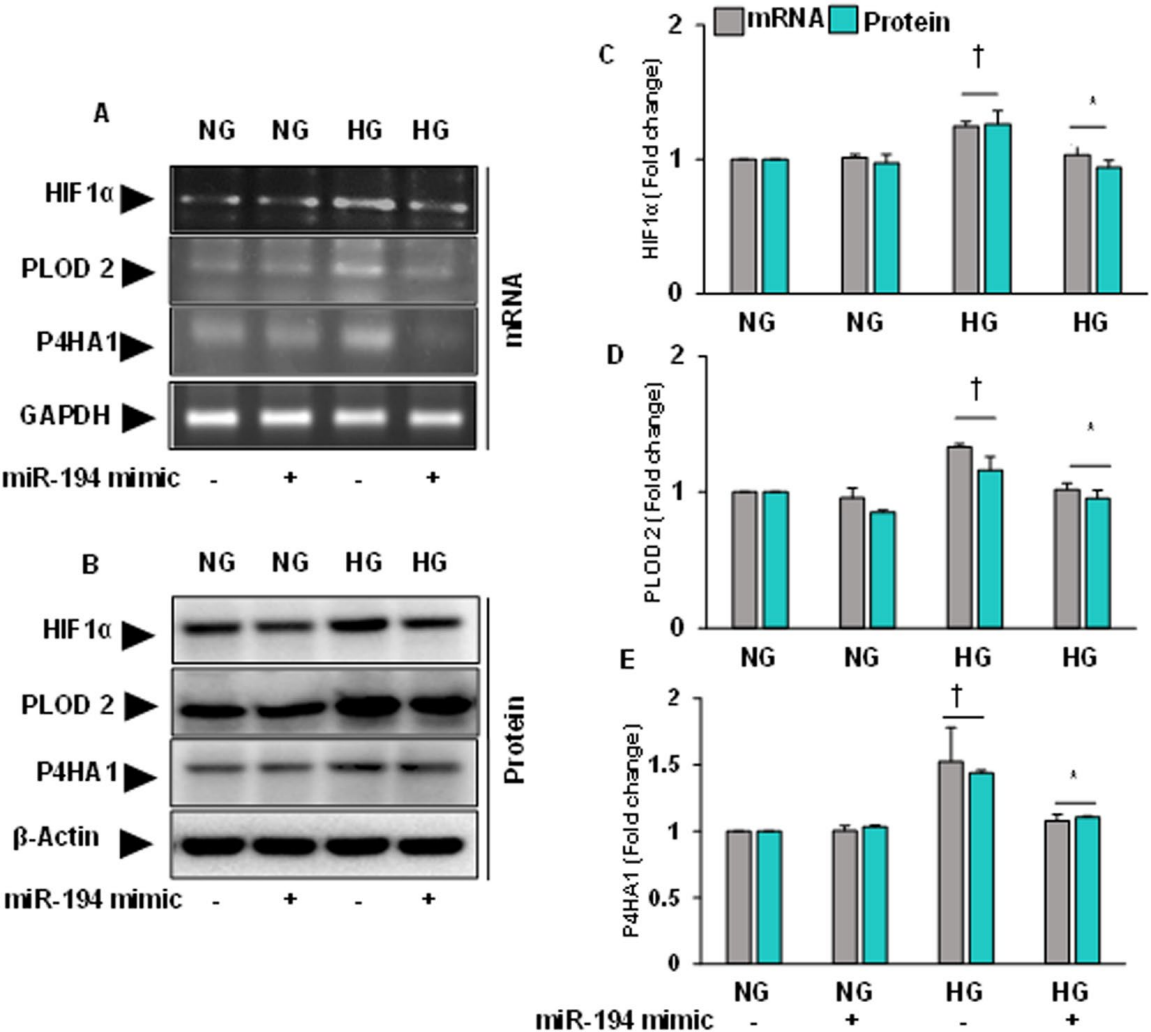
Increased expression of HIF1α, PLOD2 and P4HA1 in HG condition was normalized by miR-194 mimic transfection. **(A)** The mRNA expression was measured by RT-PCR, and **(B)** protein expression by Western blotting. **(C), (D)** and **(E)** Bar graphs representing mean fold change of HIF1α, PLOD2 and P4HA1 respectively normalized with GAPDH / β-actin. Values are mean ± SEM, n = 5 independent experiments; †p < 0.05 vs. NG alone and *p < 0.05 vs. HG alone; compared to their respective levels of mRNA and protein expression.

Regulatory role of miR-194 on HIF1α, PLOD2 and P4HA1 under HG condition was assessed by transfecting MGECs with miR-194 mimics. The results indicated that the expression of HIF1α, PLOD2 and P4HA1 was upregulated in HG condition compared to NG condition (Fig. 9A,B,C,D and E). When MGECs were transfected with miR-194 mimic, the expression of these molecules were almost normalized in HG condition compared to non-transfected cells (Fig. 9A,B,C,D and E). No significant changes were observed in NG cells without or with miR-194 mimic transfection. These results indicate that miR-194 regulates HIF1α, PLOD2 and P4HA1 in HG condition.

### Possible protein-chemical interaction and signaling involved in remodeling

**Figure 10:**
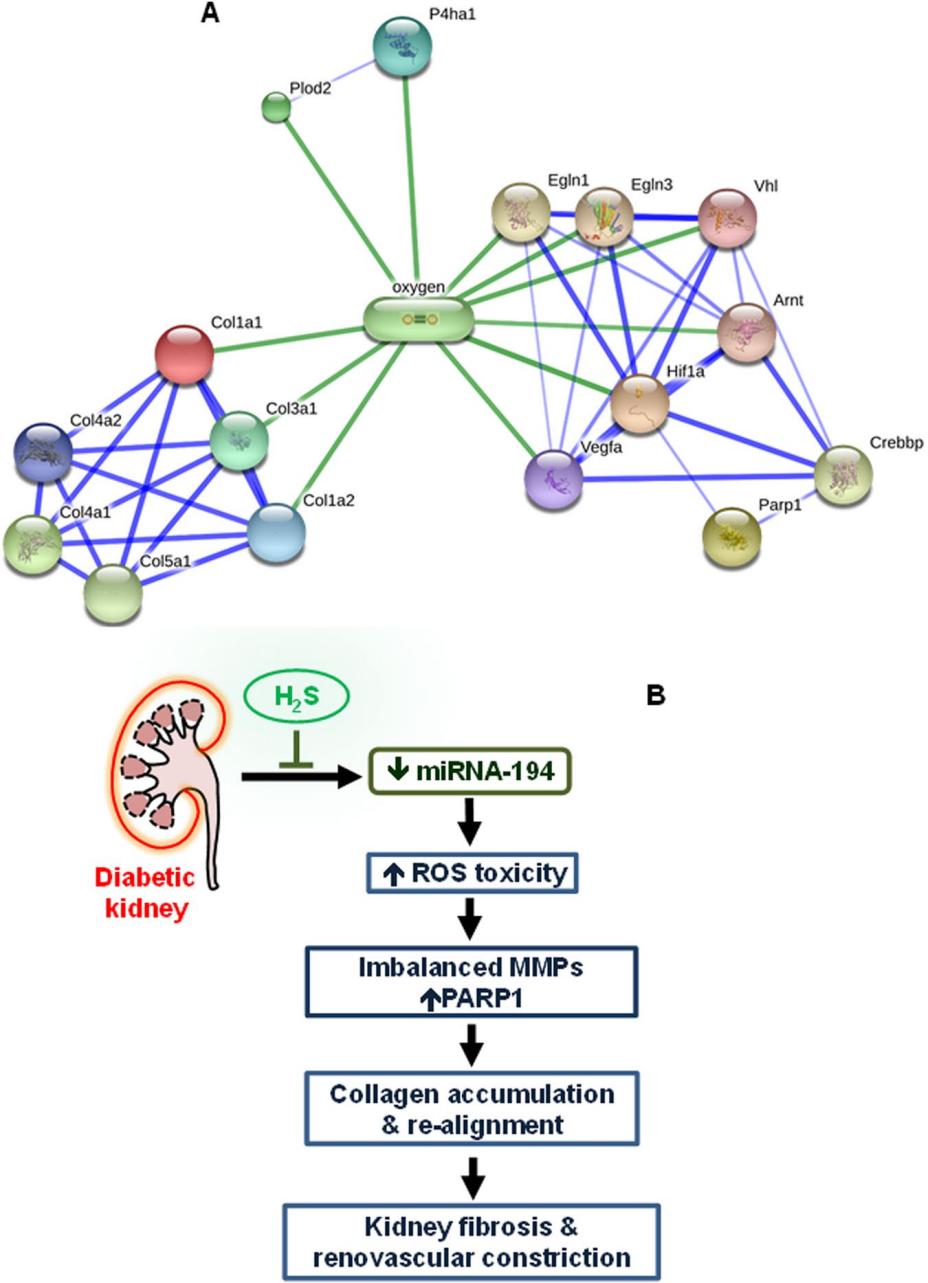
(**A)** Schematics of chemical-protein interactions: STITCH 4.0. Stronger associations are represented by thicker lines. Protein-protein interactions are shown in blue, chemical-protein interactions are in green. Abbreviations: Plod2, Procollagen-Lysine,2-Oxoglutarate 5-Dioxygenase 2; P4ha1, Prolyl 4-Hydroxylase Subunit Alpha 1; Col1a1, Collagen Type I Alpha 1 Chain; Col1a2, Collagen Type I Alpha 2 Chain; Col3a1, Collagen Type III Alpha 1 Chain; Col4a1, Collagen Type IV Alpha 1 Chain; Col5a1, Collagen Type V Alpha 1 Chain; Egln-1, Egl-9 Family Hypoxia Inducible Factor 1; Egln-3, Egl-9 Family Hypoxia Inducible Factor 3; Vhl, Von Hippel-Lindau tumor suppressor protein; Arnt, Aryl Hydrocarbon Receptor Nuclear Translocator; Hif1a, Hypoxia Inducible Factor 1 Alpha Subunit; Vegfa, Vascular Endothelial Growth Factor A; Crebbp, CAMP Responsive Element Binding Protein 1; Parp1, Poly(ADP-Ribose) Polymerase 1. **(B)** Schematic representation of central hypothesis. In diabetic kidney, diminished H_2_S and decreased miRNA-194 induces increased production of ROS. Increased ROS, in turn causes imbalance of MMPs and upregulation of PARP-1. The alterations of H_2_S, miR-194, ROS, MMPs and PARP-1 in diabetic kidney lead to collagen accumulation and re-alignment resulting in kidney fibrosis and renovascular constriction. GYY4137, a H_2_S donor, treatment normalizes miR-194 and ROS-dependent downstream pathways of renovascular remodeling, and thus preserves renovascular architecture in diabetes.

A STITCH tool was used to analyze overall obtained results from our study to better understand the molecular mechanism of remodeling process. The analysis explains protein-chemical interactions involved in molecular signaling in our study (Fig. 10A)^24^. Chemicals are represented as pill shaped, while protein in spheres. This figure showed the potential interactions between oxygen radical producing machinery; ECM components and collagen biosynthesis (Plod2, P4ha1, Col1a1, Col1a2, Col3a1, Col4a1, Col4a2 and Col5a1) and transcription / growth factors that promote vasculogenesis and angiogenesis (Egln1, Egln3, Vegfa, Hif1a, Vhl, Arnt, Crebbp) and DNA repair mechanism (Parp-1). The protein-protein binding between the three types of collagenases (Col1a1, Col1a2 and Col3a1) indicates a stronger interaction in comparison with other types. Similarly, the interactions between growth factors – Vegfa, Hif1a and Arnt are stronger which interact with the other growth factors. The synergistic action between different types of collagenases and growth factors with oxygen radical highlights the imperative establishment between these proteins and chemicals.

## Discussion

Sustained and untreated diabetes can lead to many pathological conditions causing tissue injury, kidney disease and renal fibrosis. Predominantly characterized with excess accumulation of extracellular matrix (ECM) leading to end-stage renal disease (ESRD) and kidney failure, fibrosis is regulated by various metabolic anomalies in diabetes. In recent years, an endogenous gaseous molecule H_2_S has emerged as a key physiological regulator of many metabolic and epigenetic abnormalities. Despite advances in our knowledge of H_2_S physiology in normal and disease conditions, the exact role of H_2_S in diabetic nephropathy (DN) remains obscured. The major finding from our study is that miR-194 downregulation leads to increased ROS activity in diabetic kidney. We also provide evidence of increased ROS-mediated MMPs imbalance leading to PARP1 upregulation, collagen re-alignment and accumulation of ECM. The changes in ECM components result in fibrosis and diminish vascular density in diabetic kidney. Further, our results demonstrate that supplementation of GYY (i.e., GYY4137), as a source of H_2_S ameliorates diabetic kidney remodeling through reversing miR-194 expression. Using miR-194 mimic and inhibitor, we also demonstrate that miR-194 is involved in matrix remodeling in HG condition. In Fig. 10A, we depict possible protein-protein and chemical-protein interactions, and in Fig. 10B, we highlight major findings with regulatory cascades of ECM remodeling involving miR-194 and H_2_S in diabetic condition.

In our first set of experiment, we detected a significant lower level of H_2_S in plasma of Akita mice (Fig. 1A). Following GYY treatment, the level of H_2_S was increased in Akita mice. This result is in accordance with a similar finding^11^, and suggests that GYY is effective in increasing plasma H_2_S level. We also measured blood glucose levels in our study and the results indicated that in diabetic mice following GYY treatment, blood glucose level was significantly diminished compared to non-treated diabetes group (Fig. 1B). However, the decrease was not comparable with WT control. This result suggests two possible mechanisms of GYY-mediated beneficial effects on diabetic kidney. Firstly, a direct negative effect of GYY on glucose-mediated oxidative stress, and secondly in part by reducing blood glucose level and thus stresses. In a human study, Jain and his group found a significantly lower level of H_2_S in type-2 diabetic patients compared to age matched non-diabetics^25^. Whiteman and colleagues also confirmed similar results and their findings demonstrated adiposity was negatively correlated with H_2_S levels^26^. Until now, how glucose level influences H_2_S levels and/or how H_2_S increases glucose metabolism is still unclear. Further studies are warranted to investigate the mechanisms driving these changes.

The association between reactive oxygen species (ROS) and kidney injury, both in acute and chronic, is well established^27, 28^, and an increased level of ROS is reported in diabetic conditions^29^. In addition, several studies have highlighted alterations in microRNAs are linked to stress with overproduction of ROS^30, 31^. In accordance with these previous reports, our present study demonstrated a significant increase of ROS activity and its modulator molecule ROMO1 in Akita mice (Fig. 1C,D,E and F). Interestingly, treatment with GYY decreased ROS activity which was similar to control groups, and this result was in agreement with earlier reports^17, 29, 32, 33^. The gene that encodes a mitochondrial membrane protein responsible for increased ROS is ROS modulator 1 (ROMO1). In our study, we also observed increased expression of ROMO1 in diabetic kidney which was mitigated following GYY treatment suggesting that the effect of GYY in ROS mitigation was in part due to inhibition of ROMO1 expression (Fig. 1D and F).

Both the synthesis and degradation of ECM are controlled by matrix metalloproteinase (MMPs). MMP-9 is an extensively studied MMP in the kidney, and findings from our laboratory and others have previously demonstrated an alteration of MMP-9 in fibrotic kidney diseases including diabetic nephropathy (DN)^10, 34, 35^. In the present study, we found that MMP-9 expression and activity was significantly increased in Akita kidney, which was diminished by H_2_S donor, GYY (Fig. 2A,B,C and D). We also measured the expression pattern of another fibrogenic MMP, MMP-13 in this study. We found increased expression of MMP-13 in diabetic kidney, and GYY treatment also mitigated this MMP expression (Fig. 2E and F). Previously, there were two reports on MMP-13 expression in STZ induced diabetic kidney, and the results were contradictory as one report showed a decrease^36^, while the other demonstrated an increase in its expression^37^. These differences could be due to differential pool of MMPs in the sample, i.e. urine vs. kidney. However, our results showed an increased expression of MMP-13 in genetic diabetic kidney. This findings are in line with the report by Lenz *et al*. who reported increased MMPs expression in scarring process associated with excessive degradation in the glomerulus^38^. Nonetheless, to our knowledge, our finding is the first report to demonstrate involvement of MMP-13 in genetic diabetic kidney remodeling, since Akita is a genetic diabetic mouse due to spontaneous point mutation in the *Ins2* gene.

Contrary to MMP-9 and −13 upregulation, we detected downregulation of MMP-14 in diabetic kidney, which was ameliorated by GYY (Fig. 3A and B). These results indicate a differential expression pattern of the above MMPs, which may have divergent activities in diabetic kidney remodeling. It is known that while MMP-9 is a gelatinase, MMP-13 is collagenase and MMP-14 is a membrane type MMP which specifically activate progelatinase A. Upregulation of MMPs-9 and −13 were shown to cause fibrovascular tissue formation in kidney^10, 39^, whereas downregulation of MMP-14 has been reported in the development of nephrosclerosis^40^. Thus, overall results of MMPs expression obtained from our studies are in accordance with previous reports and strongly suggest that increased expression of MMPs-9 and 13, and decreased expression of MMP-14 were involved in the development of glomerulosclerosis in diabetes. GYY reversed the expression of these MMPs in our study suggesting a possible MMPs regulatory role of H_2_S in diabetic kidney.

Activation of poly (ADP-ribose) polymerase (PARP) in diabetes and more specifically in DN is well known^41, 42^, and is correlated with increased ROS and oxidant-induced DNA injury affecting metabolic pathway and gene expression^43, 44^. Studies on PARP-1 deficient mice and with streptozotocin-induced diabetic models have indicated the involvement of PARP-1 in diabetic kidney diseases^22^. A significant increase in both the mRNA and protein expressions of PARP-1 as well as its localization in the glomerulus was evident from our study strongly supports and reaffirm the involvement of PARP-1 in diabetes (Fig. 3C,D,E and F). Further, the increased expression of PARP-1 were mitigated following GYY treatment suggesting possible regulatory mechanism of PARP-1 by H_2_S in diabetic kidney. Thus, the findings from our study suggest that GYY alleviates kidney remodeling by modulating PARP-1 in part through regulation of metabolic pathway involved in diabetes.

One of the major pathological (morphological) factors contributing to ECM turnover in diabetic glomeruli is dysregulation of collagen types I and VI. Substantial evidence from the past and recent reports have indicated increased accumulation and localization of both types of collagen (1 and VI) in diabetic glomeruli contributes for mesangial expansion^45, 46^. We found increased collagen expression in the renal tissue (Fig. 4A,B,C and D) and deposition surrounding the glomerulus of Akita mice (Fig. 5A,B and C) which was mitigated with GYY supplementation. This is in accordance with the earlier reports from our own and other labs on diabetic mice treated with H_2_S^7, 10^. Thus, the data obtained in the present study strongly suggests increased ECM turnover and GYY attenuates collagen deposition in diabetic glomeruli.

In relation to ECM turnover, recent evidences has substantially that diabetes promotes chronic hypoxia and tubulointerstitial fibrosis, a common pathway leading to ESRD^47, 48^. Hypoxia inducible factor-1 (HIF1) and more specific an oxygen-sensitive alpha (α) subunit play a crucial role towards pathological conditions including DN^47^. Studies carried out on diabetic animal models induced by Streptozotocin have demonstrated that kidney is prone to hypoxia, which occurs earlier than the development of structural changes^49^. Increased expression of HIF1α has been well documented in experimental models of renal segmental infarction and acute renal failure^50–52^. In parallel with the earlier reports, our findings in the present study have showed upregulation of HIF1α mRNA and protein levels in diabetic kidney. Following treatment with GYY, a significant decrease expression of both the gene and protein was evident diabetic kidney (Fig. 4E and G). As discussed earlier one of the significant factors, leading to fibrosis is irreversible accumulation of collagen and this is accompanied by increased pyridinoline cross-links, a hydroxylated lysine derivative. In addition, procollagen lysyl-hydroxylases (PLOD)-2 genes plays a potential role as it encodes telopeptide lysyl hydroxylase, TLH, an enzyme responsible for increased pyridinoline cross-link^53^. Additionally, studies have also shown the impact of hypoxia on these collagen forming hydroxylase enzymes regulated by PLOD2 as well as P4HA (Prolyl 4-Hydroxylase α)^54^. The same study has also highlighted the role of HIF on the synthesis of these enzymes. Our study showed an increase in both P4HA1 and PLOD2 in diabetic mice, which were mitigated by GYY treatment (Fig. 4E,F,G,H and I). These results suggest that GYY has potential in preventing collagen alignment by diminishing pyridinoline cross-link.

Vascular stiffness, constriction and compromised function is accompanied by collagen accumulation surrounding the microvascular structure. Progressive glomerular and tubulointerstitial fibrosis is characterized by increased collagen deposition and glomerular basement membrane thickening in diabetic kidney^11, 55^. Several studies have implicated inhibition or slowing down the progression of DN by H_2_S treatment through suppression of matrix protein fibronectin and collagen synthesis^11, 56^. In agreement with these previous studies, we have also detected increased collagen deposition in the peri- and glomerular space in diabetic kidney (Fig. 5A,B and C). This was halted by GYY treatment. In addition, we have also measured diminished vessel density in Akita kidney suggesting that vascular constriction was accompanied by excessive ECM deposition surrounding the kidney microvessels (Fig. 5D,E and F). Interestingly, GYY treatment ameliorated vascular density in diabetic kidney by allowing Barium sulfate penetration to the terminal microvessels, which were otherwise remained impenetrable without GYY treatment. This result along with diminished ECM deposition in diabetic kidney treated with GYY suggests that GYY not only inhibit accumulation of fibrotic collagen but also improves vascular density in part by relaxing renal microvessels.

Recent studies have highlighted the importance of microRNAs in regulating gene expression, and thus controlling many physiological activities and its aberrant expression has been shown to complicate many diseases including diabetes^57^. Expression of several miRNAs in the kidney emphasizes their critical role in the regulation of kidney development and homeostasis^58^. However, the exact role of these miRs in regulating pathophysiology of kidney diseases remains mostly unknown. Thus, unraveling miRs expression and mechanism of regulation may pave way for therapeutic strategies of many pathophysiological kidney disorders including diabetes. Studies have shown that increased expression of miR-194 in the kidney have a protective role in the prevention of diabetic renal injury^59^. On the other hand, downregulation of miR-194 in the kidney have been reported following renal ischemia-reperfusion injury (IRI)^60^. In a recent study, Jia *et al*. have demonstrated that miR-194 could be used as a potential predictor for early-stage diabetic nephropathy^61^. Our present finding is in agreement with these previous studies and demonstrated significant reduced expression of miR-194 in the diabetic kidney (Fig. 6A). Interestingly, we have also found this reduction was normalized following GYY treatment (Fig. 6A). This result highlights possible regulatory role of miR-194 by H_2_S in protecting diabetic kidney. Nonetheless, the cause and effect relationship of miR-194 downregulation and amelioration by GYY treatment in diabetic kidney was not clear from our *in vivo* studies.

Therefore, to determine the cause and effect relationships between H_2_S and miR-194, and to determine whether GYY regulates downstream molecular cascades leading to ECM deposition and remodeling in diabetic condition, we compared results using mouse glomerular endothelial cells (MGECs) treated without or with GYY along with miR-194 mimic and inhibitor in HG and NG condition. Our *in vitro* results suggest that both GYY and miR-194 mimic upregulated miR-194 expression suggesting that GYY regulates miR-194 expression in HG condition (Fig. 6B). Further to demonstrate the proof-of-concept whether miR-194 regulates oxidative stress and its modulator, MMPs, collagens and their modulating molecules in HG condition, we performed experiments as reported in Figs 7, 8 and 9. Results indicated that similar to GYY, miR-194 mimic also attenuated ROS in MGECs under HG condition (Fig. 6C). In addition, miR-194 mimic normalized ROMO-1, MMP-9, -13, -14 (Fig. 7A,B,C,D,E and F); PARP-1, Col1a, Col IV (Fig. 8A,B,C,D and E); HIF1α, PLOD2 and P4HA1 (Fig. 9A,B,C,D and E) in HG condition. These results are in agreement with our *in vivo* results of GYY-mediated molecular changes in diabetic kidney, and suggest that while GYY regulates miR-194 in diabetic kidney, mirR-194 in turn regulates these above ECM protein and their modulators in HG condition. Thus, a strong relationship of H_2_S and miR-194 exists in diabetic kidney in which GYY in concert with miR-194 modulates fibrotic vasculopathies.

It is noteworthy to mention that renal fibrosis is a multifactorial disease that involves oxidative radicals besides other known pro-fibrogenic factors^62^. Research findings from many laboratories have shown a strong relationship between miRNAs and renal fibrosis in which miRNAs regulate many mRNAs involved in diabetic nephropathy^63, 64^. Studies have also shown that miR-194 is highly expressed in the kidney^13^ and the expression level diminished in progressive CKD^65^. It is also reported that miR-194 expression was significantly reduced in both rodent and human diabetic condition^66^. In our present study, we report beneficial effect of miR-194 in diabetic renal fibrosis is in part through altered MMPs-mediated PARP-1 induction (Figs 2 and 3). We also demonstrate that pro-fibrogenic molecules i.e., MMPs and PARP-1 are the target genes of miR-194 since miR-194 mimic reverse expressed these molecules in HG condition (Figs 7 and 8). These two genes through a sequence of downstream pathways altered other pro-fibrotic genes i.e., Col1a, Col IV, HIF1α, PLOD2, P4HA1 in our experimental HG condition (Figs 8 and 9). Overall, our results suggest that the above-mentioned genes are the possible target of miR-194 in diabetic renal fibrosis.

In summary, our findings suggest that intraperitoneal injections of GYY to Akita mice alleviate diabetic renal remodeling by increasing the expression of miR-194. Increased miR-194 was accompanied with decreased ROS, MMPs-9 and -13, and PARP-1. In addition, increased accumulation and alignment of collagen was mitigated in Akita mice following GYY treatment as it was evidenced by diminished fibrosis and decrease in the expression of PLOD-1, P4HA and HIF1α. GYY treatment has further restored decreased renovascular density associated with type-1 diabetes. Thus, our findings provide evidence that in genetic diabetic kidney, H_2_S ameliorates collagen realignment and renal fibrosis, and therefore improves vascular density possibly in miR-194-dependent pathway. Regulation of miR-194 by GYY may therefore be considered as a potential target to halt or slow debilitating kidney remodeling in DN. Future in depth preclinical studies are required to confirm this initial finding on DN before GYY can be used for clinical trials and subsequent human therapy.

## Materials and Methods

### Animal Models

Mice used in this study were C57BL6/J (wild type, WT) and diabetic Akita (C57BL6/J-*Ins2* ^*Akita*^). These mice were obtained from Jackson Laboratory (Bar Harbor, ME). Animals were maintained in 12:12 h light–dark cycle with regular mouse chaw diet in the animal facility of the University of Louisville. Male mice of aged 10–14 weeks were used in this study. All animal protocols and care were carried out according to the guidelines of National Institute of Health (NIH Pub. No. 86–23, revised 1985) and were approved by the Institutional Animal Care and Use Committee (IACUC) of the University of Louisville. Animals were divided into four experimental groups: WT without GYY4137 [WT], WT treated with GYY4137 [WT + GYY], Akita without GYY4137 [Akita] and Akita with GYY4137 [Akita + GYY]. Mice were treated with GYY4137 for 8 weeks (0.25 mg/Kg/d, I.P.), while the control mice (without GYY) were given normal saline. At the end of experiment, animals were euthanized by using 2 X tribromoethanol (TBE), and both blood and kidney samples were collected.

### Antibodies and Reagents

Rabbit polyclonal antibodies against matrix metalloproteinases-9, -13, -14 and Collagen-4a were from Abcam (Cambridge, UK). Collagen-1a from Novus Biologicals (Littleton, CO). Horseradish peroxidase-linked anti-rabbit IgG antibody was from Santa Cruz Biotechnology (Santa Cruz, CA). Poly ADP ribose polymerase 1 (PARP1) and anti-GAPDH antibody were purchased from Millipore (Temecula, CA). GYY4137 was from Sigma. PVDF membrane was from Bio-Rad (Hercules, CA). Dihydroethidium (DHE) was purchased from Invitrogen (Carlsbad, CA). All other analytical reagents were from Sigma-Aldrich (Saint Louis, MO) or available highest grade.

### Total RNA and microRNA extraction

In brief, total RNA and miRNA was extracted from kidney samples using Trizol reagent (Invitrogen, Carlsbad, CA) and mirVANA™ miRNA isolation kit (Ambion, Austin, TX), respectively. Total RNA quality was determined by NanoDrop ND-1000 and RNA with high purity (260/280–2.00 and 260/230–2.00) were used for RT-PCR.

### Reverse transcription and real-time quantitative PCR

Reverse transcription was performed according to manufacturer’s protocol using Promega RT-PCR kit for the primer sequences listed in Table 1. Further, the cDNA synthesis kit from Quanta biosciences (Beverly, MA 01915) was used for miRNA. For RT-qPCR, SYBR green-based kit - EvaGreen 2× qPCR Master Mix (Bulls eye) was used to measure the relative expression of miR-194 in the presence of miRNA specific primers. Briefly, 3 steps cycling protocol was performed using 20 ng of cDNA template in a 20 μL reaction volume under the following conditions: denaturation at 95 °C for 15 min followed by 40 cycles of 94 °C for 15 s, 55 °C for 30 s, and 70 °C for 34 s in which fluorescence was acquired and detected by Roche LightCycler® 96 Real-Time PCR System (Roche Diagnostics, IN). Following RT-qPCR, analysis of melt curve was performed to validate the specific generation of the expected PCR product. SNORD68 was used as an endogenous control (Quanta Biosciences). The expression level of miR-194 was evaluated using the Roche LightCycler® 96 software version 1.1.

**Table 1:** Primer sequences of mRNAs.

mRNA	Orientation	Primers (5′–3′)
ROMO-1	Forward	*CTATGTGACCCTGCTGTG*
	Reverse	*AGAGTGCTAGGACAGTGAG*
MMP-9	Forward	*TCAGCTTAGGACAGACACCT*
	Reverse	*TGTGTACACCCACATTTGC*
MMP-13	Forward	*CAGTTGACAGGCTCCGAGAA*
	Reverse	*TTCACCCACATCAGGCACTC*
MMP-14	Forward	*CGCGCTCTAGGAATCCACAT*
	Reverse	*TTCTCATGTCCCTCCCGGAT*
PARP-1	Forward	*GTATGTCCCCCTCTTAGTCT*
	Reverse	*AGTTAGGGGTTTCAGGTAGT*
PLOD2	Forward	*CCGCAATGCTAGAGATATGA*
	Reverse	*GCTGAGCATTTGGAATGTTT*
HIF-1α	Forward	*CCCAGTTACAGAAACCTACC*
	Reverse	*TCGTTTCTTGAGGTACTTGG*
P4HA1	Forward	*GGAGCTACTGTTTTTCCTGA*
	Reverse	*ATGCCGTGTACTGTAATCTC*
Col 1a	Forward	*AGAACTTTGCTTCCCAGATG*
	Reverse	*CTATCTGTACCACCCCCTTG*
Col IV	Forward	*GACCACTATGCTTGAAGTGA*
	Reverse	*ACAGAAGGCCTTAGTAGTCT*
GAPDH	Forward	*TAAATTTAGCCCGTGTGACCT*
	Reverse	*AGGGGAAAGACTGAGAAAAC*

### Cell culture, GYY 4137 treatment and SiRNA transfection (miRNA mimics and inhibitors)

Mouse glomerular endothelial cells (MGECs) were obtained from Cell Biologics (Chicago, IL 60612). Cells were cultured and maintained in complete mouse endothelial cell medium (M1168), supplemented with growth factors at 37 °C with 5% CO_2_. Cells were seeded onto 6-well culture plates at an equal density (1 × 10^5^/well) containing either 5 mM (normal, NG) or 25 mM (high, HG) glucose for 24 hours. At 80% confluence, cells were transfected with miR-194 mimic or inhibitor using Lipofectamine RNAiMAX reagent at a final concentration of 10 nM following the manufacturer’s protocol for 48 h. We used 250 µM concentration of GYY4137 for 24 hours along with HG or NG before the cells were processed for qPCR, Western blot, DHE or immunostaining analysis.

### Plasma H_2_S and blood glucose measurement

Plasma H_2_S was measured as previously described in our publication^10^. Blood glucose was measured using commercially available kit (OneTouch Ultra2, LifeScan, Inc) following manufacturer’s instructions.

### Detection of reactive oxygen species (ROS)

ROS was detected in the kidney cryo sections and MGECs using Dihydroethidium (DHE) staining method as described previously^67^. Briefly, 5 µm thick tissue sections were fixed in ice cold acetone and MGECs were in 4% paraformaldehyde for 10 min and air dried. Sections and MGECs were incubated with DHE (10 µM/ L) in a dark humidified chamber for 10 to 15 min at room temperature. Slides were washed twice with 1x PBS and were mounted with FluoroGel mounting medium (GeneTex Inc., Irvine, CA). Images were taken by laser scanning confocal microscope (Olympus FluoView 1000, Pittsburgh, PA) and analyzed.

### Protease activity of MMP-9 by Zymography

Kidney lysate was extracted without addition of any protease inhibitors and zymography was performed as described previously with little modification^67, 68^. Briefly, kidneys were minced and placed in extraction buffer overnight at 4 °C in a shaker. After 24 hours of digestion, the extract was centrifuged and the lysate was used for gelatin zymography. Equal amount of extracted protein (25 µg) was loaded onto 10% SDS gel electrophoresis containing 0.1% gelatin. After electrophoresis, the gel was washed in 2.5% Triton X-100 for 30 min to remove SDS, then rinsed in distilled water, and incubated for overnight in activation buffer (50 mmol/l Tris.HCl, 5 mmol/l CaCl_2_, and 0.02% NaN_3_, pH 7.5) at 37 °C in a water bath with gentle agitation. Gel was then stained with 0.5% Coomassie brilliant blue R-250 in a solution containing acetic acid, methanol and water in a ratio of 1:5:4 for 1 h at room temperature. MMP activity in the gel was detected in a dark blue background with white bands.

### Western Immunoblotting

Protein was extracted from kidney tissues and cells by using RIPA buffer (Boston BioProducts, Worcester, MA, USA); with 1 mM phenylmethanesulfonyl fluoride (PMSF), and 1% protease inhibitors cocktail (Sigma, Saint Louis, MO, USA). After sonication protein lysate was centrifuged at 12,000 g for 10 min at 4 °C. Protein concentration was measured by Bradford assay. Equal amount of protein extract (25 µg) were electrophoresed by SDS-PAGE and immunoblotted onto PVDF membranes. The membranes were blocked by 5% nonfat dry milk for 1 h at room temperature and subsequently incubated overnight at 4 °C with the respective primary antibodies (1:1000). After incubation with secondary antibodies (1:5000) in room temperature, the membranes were washed and protein bands were visualized using ECL Luminata Forte (Millipore, Temecula, CA) in a Bio-Rad ChemiDoc system.

### Histological Collagen Staining (Picrosirius red)

Picrosirius red staining kit was used to detect collagen following manufacturer’s protocol and as we have reported earlier^69^. Briefly, a portion of kidney was fixed in neutral buffered formaldehyde solution. Paraffin sections of 5 µm thickness were made, deparaffinized, and hydrated in distilled water. Sections were incubated with picrosirius red stain solution for 60 minutes followed by 0.01 N HCl treatment for 2 mins. Following dehydration, sections were mounted with permount (Fisher Scientific, NJ). Slides were scanned and images were captured under light microscope. Intensity of red dye deposition in the renal cortex was measured using ImagePro plus software (Media Cybernetics, Inc. Rockville, MD).

### Immunohistochemistry (IHC)

Cryo tissue sections of 5 µm were labeled for immunofluorescence following the standard protocol. Briefly, sections were fixed in 4% paraformaldehyde and permeabilized with 0.25% Triton X-100 in PBS. Primary antibodies of MMP-14 and PARP-1 were applied and sections were incubated for overnight at 4 °C. Secondary antibodies labeled with either Alexa Fluor-488 or - 594 (Invitrogen) appropriate to the primary antibody species were applied to the sections for immunodetection of proteins. Sections were cover slipped with fluoroshield (Sigma). Stained images were visualized and analyzed for fluorescence intensity under a laser scanning confocal microscope (Olympus FluoView1000) using appropriate filter.

### Barium angiography for measuring renal vasculature

Barium sulfate was infused following the protocol as mentioned previously^10^. Briefly, the left renal artery of the mice was cannulated with PE-10 polyethelene tubing (BD, Franklin Lakes, NJ) and 1 ml of Barium sulfate at a concentration of 50 mM was injected slowly with a syringe pump. The renal vasculature was imaged by using Kodak *in vivo* imaging system FX. Software developed in the University of Lubeck, Germany was used to quantify the vessel densities. The vascular percentage was analyzed by calculating the percentage of white pixels against the pixels in the background.

### Chemical-protein interactions: STITCH 4.0

The protein-chemical interaction data were retrieved from STITCH database (Ver. 4.0) as described previously^10^.

### Statistical analysis

ImageJ software was used to calculate the mean values of protein/mRNA expression. Data were expressed as mean ± SEM of 6–7 experiments or animals/group as stated in the figure legends. Following tests for normality and homogeneity, the significance of treatment effects was determined by one-way ANOVA within and across different effectors or inhibitors. Individual comparisons between treatments were made by using Bonferroni’s multiple comparison tests using SPSS (Chicago, IL) with a significance level of *p* < 0.05.
